# Are physical activity, sedentary behaviors and sleep duration associated with body mass index-for-age and health-related quality of life among high school boys and girls?

**DOI:** 10.1186/s12955-016-0434-6

**Published:** 2016-02-27

**Authors:** Sara Jalali-Farahani, Parisa Amiri, Yit Siew Chin

**Affiliations:** Research Center for Social Determinants of Endocrine Health & Obesity Research Center, Research Institute for Endocrine Sciences, Shahid Beheshti University of Medical Sciences, Tehran, Iran; Department of Nutrition and Dietetics, Faculty of Medicine and Health Sciences, Universiti Putra Malaysia, 43400 Serdang, Selangor Malaysia; Research Centre of Excellence (RCoE), Faculty of Medicine and Health Sciences, Universiti Putra Malaysia, 43400 Serdang, Selangor Malaysia

**Keywords:** Physical activity, Sedentary behaviors, BMI-for-age, Health-related quality of life, Adolescent, Iran

## Abstract

**Background:**

Previous studies reported lower health-related quality of life (HRQOL) scores in overweight and obese adolescents compared to their normal weight counterparts; however, few studies investigated the association between obesity-related behaviors including physical activity and sedentary behaviors and HRQOL in adolescents. This study aimed at investigating the relationship between physical activity, sedentary behaviors, sleep duration and body mass index-for-age (BMI-for-age) and HRQOL among high school Tehranian students.

**Methods:**

A total of 465 high school students (48.8 % girls) were recruited from three different socio-economic zones in Tehran. The BMI-for-age was determined and physical activity and HRQOL were assessed using validated questionnaires including Quantification de l’Activite Physique en Altitude Chez les Enfants (QAPACE) and Pediatric Quality of Life Inventory (PedsQL) respectively.

**Results:**

Over one third of students (38.5 %) were either overweight or obese. The means of all self- and parent-reported HRQOL scores were significantly lower in girls, compared to boys, except for the parent-reported social functioning subscale. Mean hours of daily sleeping were significantly higher in girls, compared to boys (8.16 ± 1.27 vs. 7.73 ± 1.22 respectively; *p* < 0.05). Both girls and boys spent more time on sedentary activities than engaging in sport activities. During school and vacation periods, boys had significantly higher daily energy expenditure (DEE) compared to girls (*p* < 0.05). Only DEE during school period had a significant inverse correlation with BMI-for-age in boys but not girls (r = −0.14, *p* < 0.05). In addition, self-reported HRQOL scores were significantly associated with weekly hours adolescents spent on videogames/internet, listening to music and reading, watching TV, sports as well as DEE through sex-specific patterns. However according to parents’ perspective only weekly hours spent on listening to music and readings and sport activities were significantly associated with their children HRQOL scores.

**Conclusion:**

In summary, time spent on physical and sedentary activities were not associated with BMI-for-age, although both of these were associated with the HRQOL of high school students. The potential role of sedentary activities and physical activity should be considered in future interventions aimed at improving HRQOL in adolescents.

## Background

Physical activity among adolescents is known to be associated with both the physical and mental aspects of health [1–3]. Based on evidence, obesity, a risk factor of several chronic diseases, is associated with unhealthy behaviors including inadequate physical activity and increased sedentary behaviors in adolescents [4, 5]. Previous studies report that the amount of time spent in a vehicle, sitting in front of the television, and playing electronic games are positively related to overweight and obesity [6, 7]. Sedentary behaviors have been associated with obesity as a result of replacement with physical activity, decrease in metabolic rate or increase in food intake and consequently energy intakes [8–10]. Besides, sleep duration is another factor that seems to be associated with overweight and obesity. While there is evidence suggesting that short sleep duration was related to weight gain and obesity in children [11], there is inconsistent data regarding the association between shortened sleep duration and obesity in adolescents [12].

In addition to the association between physical activity, sedentary behaviors and obesity, there are several studies showing the influence of physical activity and sedentary behaviors on different aspects of mental health in adolescents [1, 13, 14]. Most existing studies regarding associations between physical activity and health in Iranian adolescents focused mainly on certain dimensions of health [15, 16]. Based on the world health organization (WHO) definition for health, the health-related quality of life (HRQOL) is a multidimensional construct that includes the three main dimensions of health (physical, psychological and social) and can hence serve as an outcome measure of overall health. In this context, limited studies have investigated physical activity and sedentary behaviors in relation to HRQOL in adolescents [17–19]; unfortunately their data are limited to developed societies and there is lack of evidence on this association in Iranian adolescents. Considering the childhood obesity epidemic [20] and the lack of evidence regarding the association between physical activity and HRQOL in high school Iranian students, the current study aims to assess physical activity, sedentary behaviors and sleep duration in relation to body mass index-for-age (BMI-for-age) as an objective measurement of their health and HRQOL as a subjective measurement of their health.

## Methods

### Participants

This was a cross-sectional study conducted among high school students (14–17 years old) residing in Tehran. Participants were selected using multi-stage sampling method from three diverse geographical zones (North zone, Central zone and South zone) of Tehran with different socio-economic backgrounds. After randomly selecting one district in each zone, two high schools (one female high school and one male high school) were randomly selected from each district. Since, the Iranian high school has a duration of three school years, one class was randomly selected from each school year (a total of three classes were selected from each high school) and all students of these classes were invited to participate in this study. Those students who refused to participate in the study, had any chronic diseases, or had incomplete data were excluded from the study. Of a total of 485 students invited to participate in the study, two students (one girl and one boy) refused to participate in the study, four students had chronic diseases and another 14 students did not return the parental questionnaire to the researchers; hence, a total of 20 students were excluded from the study and the remaining data of 465 students were analyzed.

### Measurements

Body weight and height of the students were measured by trained researchers. The World Health Organization (WHO) AnthroPlus (version 3.2.2) and macros software were used to determine BMI-for-age. Body weight status of the students was determined using WHO Growth References (2007) [21]. In addition, to assess physical activity and health-related quality of life, students were asked to complete self-administered questionnaires, including the Quantification de l’Activite Physique en Altitude Chez les Enfants (QAPACE) and Pediatric Quality of Life Inventory (PedsQL^TM^) respectively [22, 23]. The QAPACE, is a self-administered questionnaire consisting of 18 items that assess 13 categories of daily physical activities, which include: 1) sleeping, 2) toilet, 3) meals, 4) transportation, 5) classroom activities and homework, 6) mandatory physical education, 7) other activities in school, 8) out of school activities, 9) religious activities, 10) vacation activities, 11) artistic activities, 12) competition sports and 13) domestic activities in home. The questionnaire covers all possible school or vacation activities of adolescents over the past year and can hence be used to measure the mean daily energy expenditure (DEE) over the past year, during school and vacation, 24 h a day [22]. According to the original questionnaire, information about activities during school and vacations, along with the physical activity compendium [24] were used to calculate the annual mean DEE, using the formula:

$$ \begin{array}{l}\kern3.25em \mathrm{i}=13\kern0.75em \\ {}\mathrm{D}\mathrm{E}\mathrm{E}=\varSigma \kern0.5em \left(\left(\left(\left({\mathrm{f}}_{\mathrm{sp}}\kern0.5em \left(\mathrm{i}\right).\ {\mathrm{d}}_{\mathrm{sp}}\ \left(\mathrm{i}\right).\ 258\right) + \left({\mathrm{f}}_{\mathrm{vp}}\ \left(\mathrm{i}\right).\ {\mathrm{d}}_{\mathrm{vp}}\ \left(\mathrm{i}\right).\ 107\right)\right)/365\right)\ \mathrm{m}\ \left(\mathrm{i}\right)\right)\\ {}\kern3.5em \mathrm{i}=1\end{array} $$

The sum is extended over all possible activities i (i = 1 to 13 categories). For each activity f(i), d(i) and m(i) correspond to its daily frequency, mean duration and intensity respectively according to the physical activity compendium, SP and VP correspond to school period and vacation period respectively [22]. Before using this questionnaire for the current study, the QAPACE questionnaire was translated into Persian, and the reliability and validity of the Iranian version was tested, with results showing acceptable reliability and validity [25].

The PedsQL^TM^ is a self-administered questionnaire that includes child-self report and parent-proxy report, both of which encompass 23 items and four subscales (Physical Functioning, Emotional Functioning, Social Functioning, and School Functioning). Both reports were scored on a five-point scale from 0 (never a problem) to 4 (almost always a problem) by the respondents. For ease of interpretability, the 0–4 scale items are transformed to 0–100 as follows: 0 = 100, 1 = 75, 2 = 50, 3 = 25, 4 = 0; hence, higher scores indicate better HRQOL [23]. Reliability and validity of the Iranian version of PedsQL have been reported previously [26].

To obtain information on socio-demographics and parental perception of student’s HRQOL, the students passed on the parent-version questionnaire (including socio-demographic information and parent proxy-report of Pediatric Quality of Life Inventory (PedsQL)) to their parents for completion, following which the parent-version questionnaire was returned to the researcher via students.

### Ethics

Ethic approvals were obtained from Medical Research Ethical Committee of the Faculty of Medicine and Health Sciences, Universiti Putra Malaysia (UPM) in Malaysia and the Research Institute for Endocrine Sciences of Shahid Beheshti University of Medical Sciences in Iran. Written informed consent was obtained from both students and their parents.

### Statistical analysis

Data were statistically analyzed using SPSS software (version 19.0). The Independent samples *T*-test was used to compare continuous variables between boys and girls. To determine the correlation between continuous variables, Pearson correlation coefficients were reported; for not normally distributed data, equivalent nonparametric tests were performed. Statistical significance was set at *p* < 0.05.

## Results

A total of 465 high school students (48.8 % girls), aged 14–17 y participated in this study. Over one third of students (38.5 %) were either overweight or obese, 58.7 % were normal weight and 2.8 % were severely thin or thin. The means of self-reported HRQOL scores were significantly lower in girls, compared to boys (Physical functioning: 80.78 ± 13.50 vs. 89.26 ± 10.03, emotional functioning: 62.89 ± 19.97 vs. 76.45 ± 14.62, social functioning: 85.07 ± 16.72 vs. 89.37 ± 11.42, school functioning: 73.26 ± 15.45 vs. 78.42 ± 16.17 and total HRQOL scores: 76.19 ± 12.93 vs. 84.14 ± 9.73 in girls and boys respectively; *p* < 0.05). Similarly, according to parent proxy-reports, all subscale and total scores of HRQOL were significantly lower in girls, compared to boys (Physical functioning: 82.08 ± 16.56 vs. 88.99 ± 13.34, emotional functioning: 65.22 ± 24.21 vs. 76.53 ± 18.05, school functioning: 76.28 ± 17.42 vs. 79.85 ± 17.32 and total HRQOL scores: 78.41 ± 15.30 vs. 84.03 ± 12.24 in girls and boys respectively; *p* < 0.05) except for the social functioning subscale scores (Table 1).

**Table 1:** Descriptive statistics of age, BMI-for-age, body weight status and health-related quality of life (HRQOL) scores of students by gender

	Girls (*n* = 227)	Boys (*n* = 238)	*P* value
Age (years)	15.45 ± 0.96	15.66 ± 0.91	0.018*
BMI-for-age	0.47 ± 1.28	0.47 ± 1.45	0.964
Body weight status *n* (%)			
Severely thin and thin	3(1.3 %)	10(4.2 %)	0.125
Normal weight	143(63.0 %)	130(54.6 %)	
Overweight	55(24.2 %)	67(28.2 %)	
Obese	26(11.5 %)	31(13.0 %)	
Adolescent self-report of HRQOL scores			
Physical functioning	80.78 ± 13.50	89.26 ± 10.03	<0.001*
Emotional functioning	62.89 ± 19.97	76.45 ± 14.62	<0.001*
Social functioning	85.07 ± 16.72	89.37 ± 11.42	0.025*
School functioning	73.26 ± 15.45	78.42 ± 16.17	<0.001*
Total HRQOL score	76.19 ± 12.93	84.14 ± 9.73	<0.001*
Parent proxy-reports of HRQOL scores			
Physical functioning	82.08 ± 16.56	88.99 ± 13.34	<0.001*
Emotional functioning	65.22 ± 24.21	76.53 ± 18.05	<0.001*
Social functioning	87.73 ± 16.31	87.79 ± 14.88	0.498
School functioning	76.28 ± 17.42	79.85 ± 17.32	0.027*
Total HRQOL score	78.41 ± 15.30	84.03 ± 12.24	<0.001*

*p < 0.05

Table 2 indicates, mean weekly times spent on different activities and energy expenditure during school and vacation periods. The mean hours of daily sleeping were significantly higher in girls, compared to boys (8.16 ± 1.27 vs. 7.73 ± 1.22 respectively; *p* < 0.05), students spent more time on sedentary activities (20.8 ± 9.7 and 27.8 ± 11.1 h/week during school and vacation periods respectively) than engaging in sport activities (1.5 (0.5-3.75) and 4.8 ± 4.2 h/week during school and vacation periods respectively). During school periods, the mean weekly time spent on both screen time viewing (watching TV and Videogames/Internet) and on sport activities were significantly higher in boys, compared to girls (screen time viewing: 12.87 ± 6.35 vs. 10.44 ± 6.5 h/week, sport activities: 3.0(1.0-5.0) vs. 1.0(0–2.3) hour/week in boys and girls respectively; *p* < 0.05). During vacation periods, the mean weekly time spent on playing video games/internet and sport activities were significantly higher in boys, compared to girls (video games/internet: 8.18 ± 4.96 vs. 6.26 ± 5.11 h/week, sport activities: 5.86 ± 4.30 vs. 3.74 ± 3.77 h/week in boys and girls respectively; *p* < 0.05), while the mean weekly time spent on listening music/reading was significantly higher in girls, compared to boys (13.02 ± 6.65 vs. 8.86 ± 6.48 h/week respectively; *p* < 0.05). During both school and vacation periods, boys had significantly higher DEE, compared to girls (*p* < 0.05).

**Table 2:** Descriptive statistics of duration of time spent by high school students on selected activities and their daily energy expenditure (DEE)

	Girls (*n* = 227)	Boys (*n* = 238)	*P* value
Sleeping (hours/day)	8.16 ± 1.27*	7.73 ± 1.22	<0.001^**^
Sedentary and sport activities during school period			
Screen time viewing (hours/week)	10.44 ± 6.5	12.87 ± 6.35	<0.001^**^
-Watching TV (hours/week)	7.39 ± 4.45	8.36 ± 4.14	0.015^**^
-Videogames/Internet (hours/week)	1.5(0.5-3.8)	3.5(1.0-6.4)	<0.001^**^
Listening to music/reading (hours/week)	9.62 ± 6.49	8.58 ± 6.62	0.087
Sport (hours/week)	1.0(0–2.3)	3.0(1.0-5.0)	<0.001^**^
Sedentary and sport activities during vacation period			
Screen time viewing (hours/week)	15.87 ± 7.63	17.95 ± 7.32	0.003^**^
-Watching TV (hours/week)	10.5(6.0-14.0)	10.5(7.0-14.0)	0.789
-Videogames/Internet (hours/week)	6.26 ± 5.11	8.18 ± 4.96	<0.001^**^
Listening to music/reading (hours/week)	13.02 ± 6.65	8.86 ± 6.48	<0.001^**^
Sport (hours/week)	3.74 ± 3.77	5.86 ± 4.30	<0.001^**^
Daily energy expenditure			
DEE during school period (kcal/day)	1853(1543–2252)	2280(1863–2880)	<0.001^**^
DEE during vacation period (kcal/day)	1244(995–1660)	1594(1219–2199)	<0.001^**^
DEE total year (kcal/day)	1660(1402–2061)	2113(1673–2710)	<0.001^**^
DEE during school period per kg body weight (kcal/kg.day)	31.0(28.1-35.3)	31.8(28.2-40.9)	0.020^**^
DEE during vacation period per kg body weight (kcal/kg.day)	20.8(17.8-26.1)	23.1(17.7-32.3)	0.037^**^
DEE total year per kg body weight (kcal/kg.day)	28.0(25.1-32.6)	29.1(25.4-38.0)	0.003^**^

*Data are presented as mean ± sd or median (IQR)

**p < 0.05

The correlations between time spent on different activities and BMI-for-age as well as DEE and BMI-for-age are presented in Table 3; as indicated, there were no significant correlations between duration of weekly hours spent on different activities and BMI-for-age in both girls and boys (r ranged from 0.01-0.09); however in boys, DEE during school periods had a significant inverse correlation with BMI-for-age (*r* = −0.14, *p* < 0.05), whereas among girls, DEE was not significantly correlated to BMI-for-age during school and vacation periods, (*r* = 0.04 for both).

**Table 3:** Correlation coefficients (r or r_s_) for correlation between duration of time spent on sleeping, selected categories of activities, daily energy expenditure (DEE) and BMI-for-age

	Girls (*n* = 227)	Boys (*n* = 238)
Sleeping duration (hours/day)	0.02	0.01
Sedentary and sport activities during school period		
Screen time viewing (hours/week)	0.01	0.06
-Watching TV (hours/week)	0.01	0.09
-Videogames/Internet (hours/week)	−0.03	−0.03
Listening to music/reading (hours/week)	−0.04	0.01
Sport activities (hours/week)	0.02	−0.09
Sedentary and sport activities during vacation period		
Screen time viewing (hours/week)	0.06	0.07
-Watching TV (hours/week)	0.07	0.09
-Videogames/Internet (hours/week)	0.04	0.03
Listening to music/reading (hours/week)	0.06	0.02
Sport activities (hours/week)	0.03	−0.03
DEE during school period per kg body weight (kcal/kg.day)	0.04	−0.14*
DEE during vacation period per kg body weight (kcal/kg.day)	0.04	−0.06
DEE total year per kg body weight (kcal/kg.day)	0.04	−0.11

*p < 0.05

Table 4 indicates the correlation between duration of time spent on sleeping, selected categories of activities, DEE and self-reported HRQOL scores in girls and boys. There were no significant correlations between sleeping duration and HRQOL subscale and total scores. In terms of time spent on different activities during school periods, in boys, weekly videogames/internet duration was inversely correlated to school functioning score indicating that time spent on playing video game and surfing the net was related to poor school functioning in this group. Besides, weekly hours spent on listening to music and reading were significantly correlated to social functioning scores in girls and physical functioning scores in boys; also weekly hours spent on sports were significantly correlated to all subscale and total scores of HRQOL in girls and physical and social functioning subscale scores in boys. During vacation periods, there were significant correlations between weekly time spent on watching TV and social functioning score and weekly time spent on listening to music/reading and emotional functioning in boys. On the other hand, weekly time spent on sport activities was significantly correlated to social functioning score in girls and physical functioning, emotional functioning, social functioning and total HRQOL scores in boys. In addition, there were significant correlations between DEE and social functioning scores in girls as well as DEE and physical functioning, social functioning and total HRQOL scores in boys.

**Table 4:** Correlation coefficients (r or r_s_) for correlation between duration of time spent on sleeping, selected categories of activities, daily energy expenditure (DEE) and self-reported health-related quality of life (HRQOL) scores

	Girls (*n* = 227)					Boys (*n* = 238)				
	Physical	Emotional	Social	School	Total score	Physical	Emotional	Social	School	Total score
Sleeping duration (hours/day)	−0.01	−0.01	−0.04	−0.06	−0.02	−0.04	0.06	0.01	0	0.01
Sedentary and sport activities during school period										
Screen time viewing (hours/week)	0.05	0.12	0.04	0.01	0.09	0.05	−0.05	−0.01	−0.15*	−0.05
-Watching TV (hours/week)	0.06	0.09	0	0.02	0.06	−0.01	−0.05	−0.09	−0.10	−0.07
-Videogames/Internet (hours/week)	0	0.12	0.09	0.07	0.09	0.07	0	0.05	−0.16*	−0.03
Listening music/reading (hours/week)	−0.04	−0.06	0.14*	−0.05	−0.01	0.13*	−0.12	0.02	0.09	0.05
Sport activities (hours/week)	0.22*	0.16*	0.23*	0.17*	0.24*	0.20*	0.04	0.16*	0	0.11
Sedentary and sport activities during vacation period										
Screen time viewing (hours/week)	0.02	0.06	−0.04	0.07	0.03	0.03	−0.05	−0.10	−0.04	−0.05
-Watching TV (hours/week)	0	0.01	−0.11	0	−0.03	0	−0.04	−0.13*	−0.01	−0.05
-Videogames/Internet (hours/week)	0.05	0.09	0.05	0.10	0.09	0.02	−0.05	−0.03	−0.04	−0.03
Listening music/reading (hours/week)	−0.01	0.03	0.11	0.09	0.06	0.11	−0.16*	0.01	0.06	0.01
Sport activities (hours/week)	0.09	0.09	0.26*	0.02	0.11	0.25*	0.13*	0.16*	0.11	0.21*
DEE total year per kg body weight (kcal/kg.day)	0.12	0.04	0.20*	0.03	0.12	0.19*	0.05	0.17*	0.08	0.14*

*p < 0.05

Table 5 displays the correlations between duration of time spent on sleeping, selected categories of activities, DEE and HRQOL scores in girls and boys according to parents’ perspectives. There were no significant association between sleeping duration and subscales and total scores of HRQOL. During school periods, weekly hours spent on sport activities was significantly correlated to physical functioning score in girls and inversely correlated to school functioning in boys. During vacation periods, weekly time spent on sport activities was significantly correlated to physical and social functioning scores in girls and weekly time spent on listening to music/reading was inversely correlated to emotional functioning in boys. Furthermore, there were no significant correlations between DEE and parent-reported HRQOL scores.

**Table 5:** Correlation coefficients (r or r_s_) for correlation between duration of time spent on sleeping, selected categories of activities, daily energy expenditure (DEE) and parental reported health-related quality of life (HRQOL) scores

	Girls (*n* = 227)					Boys (*n* = 238)				
	Physical	Emotional	Social	School	Total score	Physical	Emotional	Social	School	Total score
Sleeping duration (hours/day)	−0.07	0.02	−0.04	−0.07	0.03	−0.02	0.09	0.06	0.05	0.06
Sedentary and sport activities during school period										
Screen time viewing (hours/week)	0.05	0.07	0.11	0.01	0.10	−0.01	−0.04	0.01	−0.06	−0.03
-Watching TV (hours/week)	0	0.07	0.05	0.03	0.05	−0.02	−0.03	−0.03	−0.05	−0.03
-Videogames/Internet (hours/week)	0.05	0.07	0.11	0.05	0.10	0.07	−0.02	0.02	−0.09	−0.05
Listening music/reading (hours/week)	−0.08	−0.08	0.08	−0.07	−0.08	−0.03	−0.08	0.01	−0.08	−0.03
Sport activities (hours/week)	0.15*	0.01	0.10	0.05	0.07	0.10	0.01	0.04	−0.14*	−0.02
Sedentary and sport activities during vacation period										
Screen time viewing (hours/week)	−0.03	0.07	−0.05	0.02	0	−0.02	−0.06	−0.07	0	−0.05
-Watching TV (hours/week)	−0.04	0.07	−0.07	0.01	0.01	0.01	−0.04	−0.06	−0.01	−0.02
-Videogames/Internet (hours/week)	0	0.04	0	0.03	0.02	−0.05	−0.05	−0.05	−0.01	−0.03
Listening music/reading (hours/week)	−0.06	−0.07	0.04	−0.07	−0.05	−0.01	−0.14*	−0.03	−0.01	−0.04
Sport activities (hours/week)	0.15*	0.03	0.17*	0.03	0.08	0.03	0.06	0.09	−0.03	0.09
DEE total year per kg body weight (kcal/kg.day)	0.02	−0.04	0.08	−0.04	−0.07	0	0	0.03	−0.10	−0.03

*p < 0.05

## Discussion

In the current study, the mean hours of daily sleeping was significantly higher in girls, compared to boys. In both girls and boys, the average time spent on sedentary activities by the students was much higher than the time allocated to sport activities. Furthermore, during both school and vacation periods, the mean weekly time spent on sport activities was significantly higher in boys, compared to girls, findings similar to those of studies reporting that Iranian adolescent boys spent more time on exercising and physical activity as compared to girls [27–29]. Furthermore, time spent on screen time viewing during school periods and time spent on playing videogames and surfing the net during vacation periods were significantly higher in boys compared to girls. Previous studies also reported gender differences in the average time spent on video games [30, 31], as reported by Homer et al. [30] who found that preadolescent boys spend more time (about 43 h/week) on playing video games, compared to girls (about 30 h/week) [30]; these findings are in agreement with those of the current study and a previous study conducted in Iran [29]. However, the mean weekly time spent on listening to music/reading was significantly higher in girls, compared to boys during vacation periods . In this study boys had significantly higher DEE compared to girls, in agreement with findings of previous studies [32, 33].

In the current study, no significant association was found between weekly time spent on different activities and BMI-for-age. In addition, there was no significant correlation between DEE and BMI-for-age during vacation and school periods in both sexes, except for DEE per kilogram of body weight, which was inversely correlated to BMI-for-age in boys during school periods. Previous studies reported higher total energy expenditure (TEE) in obese children and adolescents, compared to their non-obese counterparts [32, 34]. In line with these findings, Hallal et al. [35] reported no significant cross-sectional or longitudinal associations between physical activity and body composition in adolescents [35]. In a systematic review conducted by Wilks et al. (2010), physical activity was not strongly associated with changes of fat mass in children [36]. The aforementioned studies, do not support the association between physical activity and body weight in adolescents, which may be due to the fact that obesity is the result of imbalance between energy intake and energy expenditure, and both important components of energy balance should be considered simultaneously [37]. As reported by Luke and Cooper [38], considering the limited empirical evidence available supporting the effectiveness of physical activity in prevention and treatment of obesity, other influencing factors such as dietary intakes and food environment need to be explored [38]. However, these findings do not deny the important role of physical activity in promotion of overall health; this study also supports the association between physical activity and HRQOL in adolescents which is discussed below.

Sleeping duration was not correlated to any of the HRQOL subscales and total scores in the current study. In terms of time spent on different activities during school periods, the current study findings indicate that spending more time on playing video game and surfing internet was related to poor school functioning in boys, and spending more time on listening to music/reading and sport activities were related to better HRQOL scores. During vacation periods, spending more time on watching TV and listening to music/reading were related to poorer HRQOL in boys. Whereas, spending more time on sport activities was correlated to better HRQOL scores in both sexes. Overall, time spent on screen time activities was related to poorer HRQOL in boys, while in both adolescent boys and girls, time spent on physical activity was related to better HRQOL scores specifically in physical and social subscales. Regarding time spent on listening to music/reading, a different pattern was observed; during school periods, it was related to better HRQOL in the social dimension in girls and in the physical dimension in boys, while during vacation periods it was related to poor emotional HRQOL scores in boys. Previous studies indicate that lifestyle factors were associated with HRQOL in children and adolescents [18, 19, 39–43]; however, the data available are mainly from developed countries, while evidence from developing countries on this association is scarce, highlighting the current study as being one among the first from a developing country. In terms of the association between physical activity and HRQOL, most existing studies show that physical activity was related to better HRQOL [19, 39–41, 43]. However, findings of another study conducted by Boyle et al. [44] indicated that physical activity was not significantly associated with HRQOL of British adolescents (*n* = 1771), aged 11–15 years. Inconsistencies in the aforementioned findings may be due to using different methods for measurement of physical activity. In terms of screen time viewing, most findings of previous studies [18, 39, 41, 43] demonstrated that time spent on watching TV, playing video games and surfing the net was inversely associated with HRQOL scores in children and adolescents, results in line with our findings in boys. Since most of the studies mentioned had a cross-sectional nature, causal association cannot be determined. Clinical trials and prospective research are needed to determine whether low physical activity and high sedentary activities can result in poor HRQOL in adolescents or vice versa. In this context, clinical trial studies exist which support the potential role of physical activity in improving HRQOL of adolescents [45, 46].

In this study, similar to findings from the self-reported HRQOL data of adolescents, there was no association between sleeping duration and HRQOL subscales and total scores according to parents’ perspectives. Furthermore, based on parents’ perspectives, the time spent on sport activities during school and vacation periods were related to better scores of HRQOL in physical and social domains in girls, which is similar to adolescents’ reports. In boys however, time spent on sport activities during school periods was not related to HRQOL in boys except for a significant correlation to poorer HRQOL in school functioning, a finding not in agreement with adolescents’ perspectives. No significant association was found between time spent on sedentary activities and HRQOL total and subscales scores, except for an inverse correlation between time spent on listening to music/reading and the emotional dimension of HRQOL in boys. Findings of previous research regarding associations between physical activity, sedentary activities and HRQOL were mostly based on the self-reported HRQOL data by adolescents [18, 40, 41, 43, 44] and only one study included parents’ perspectives [47]. Vella et al. [47] reported a longitudinal association between participating in sport activities and parental reported HRQOL among children, aged 8–10 years; their findings indicated better HRQOL at age 10 for children who maintained participation in sports between 8 to 10 years of age, compared to those who had either never participated in sports, had dropped out of sport programs during this period or commenced participation in sport programs at 2-year follow up [47].

As one of its major strengths, this study makes up for the limitations of previous studies that examined only the association between physical and sedentary activities and certain dimensions of health. In addition, in the current study both adolescents’ and parents’ perspectives were obtained regarding HRQOL and views of both groups were analyzed and compared while previous studies only reported either the adolescents’ or the parents’ perspectives. Besides, using the QAPACE questionnaire provided the chance to obtain physical activity data during school and vacation periods separately. The limitations of this study should also be taken into account. First, since the study was cross-sectional we could not make causal inferences about relationships between studied variables. Second, recruiting a sample from high school students residing in an urban area of Tehran, reduced the generalizability of these findings to other parts of the country. Furthermore, in the current study, we used self-reported rather than objective measurements of physical activity and sedentary activities; hence, difficulty in estimation and recalling durations of activities by students may have reduced the validity of self-reported data on activity.

## Conclusion

In summary, the findings of this study indicate that time spent on physical activity and sedentary activities was not associated with BMI-for-age; however, both these were associated with HRQOL in high school students especially in the physical and psychosocial domains. Considering the potentially positive role of physical activity and negative role of sedentary activities on HRQOL of high school students, related health professionals should incorporate physical activity and sedentary activities into future policies and intervention programs aimed at improving HRQOL in adolescents.
